# An Ambulatory Approach to Atrioventricular Block in Lyme Carditis Using Temporary Permanent Pacemakers

**DOI:** 10.19102/icrm.2023.14033

**Published:** 2023-03-15

**Keywords:** Atrioventricular block, Lyme carditis, temporary permanent pacemaker

We read with interest the case described by Aromin et al. on the use of an ambulatory approach to the treatment of high-degree atrioventricular (AV) block (AVB) secondary to Lyme carditis (LC).^1^ The patient described is a 31-year-old previously healthy man who presented with syncope, fever, and erythema migrans weeks after a witnessed tick bite while camping in the Lyme-endemic region of Ontario. With a high Suspicious Index in Lyme Carditis (SILC)^2^ score of 12 points, he was at high risk for LC and started on intravenous (IV) ceftriaxone, admitted for continuous cardiac monitoring, and supported with a temporary transvenous pacemaker (TTVP). As his pacing burden remained high 5 days post-admission and his presentation coincided with the peak of the coronavirus disease 2019 (COVID-19) pandemic, he received a temporary permanent pacemaker (TPPM) and was discharged home to complete a 21-day course of IV ceftriaxone. Finally, after a full course of antibiotics, the patient underwent a stress test, which showed adequate 1:1 AV conduction, and the TPPM was successfully removed.

It is important to note that Aromin et al.’s management of LC with high-degree AVB was consistent with our recommended approach.^3^ This includes (1) use of the SILC score to determine the risk of LC; (2) early initiation of antibiotics; (3) avoidance of PPMs, instead, using a TPPM as supportive management of symptomatic heart block; and (4) use of a stress test ≥10–14 days after initiating IV antibiotics to ensure 1:1 AV conduction at a heart rate of >120 bpm. While the patient’s symptomatic heart block was initially managed with a TTVP, necessitating bed rest and admission to the coronary care unit, the recommended approach is to use TPPM at the outset for all patients suspected of having LC necessitating pacing due to symptomatic bradycardia.^4^

As the duration of AVB in LC varies and can last for several days after the initiation of antibiotic therapy, TPPM offers a stable form of ventricular pacing support while promoting early patient mobilization.^4^ Previous studies have demonstrated that TPPM use is associated with decreased rates of lead dislocation and complications (eg, severe bradycardia requiring resuscitation, infection, inappropriate pacing, and venous thrombosis) compared to traditional TTVPs.^5^ Our single-center experience in the Lyme-endemic region of southeastern Ontario shows that, of the 21 patients diagnosed with LC at our center in the last 5 years, 4 patients received TPPMs for the management of symptomatic bradycardia. The average duration of TPPM implant was 10.5 (standard deviation, 1.9) days, and there were no procedure-related complications. On long-term follow-up, all patients had resolution of conduction abnormalities without a need for permanent pacing.^6^

Finally, while we usually recommend daily assessment of patients with TPPMs, we recognize that the unique overlap of this patient presentation with the height of the COVID-19 pandemic necessitated novel strategies for rapid patient turnover and discharges. The authors clearly made efforts to identify the ideal patient for this ambulatory approach: a patient who is otherwise healthy, highly engaged, reliable, and lived near the hospital.^1^

Chang Nancy Wang, md^1^ and Adrian Baranchuk, md, facc, frcpc, fccs (adrian.baranchuk@kingstonhsc.ca)^1^

^1^Division of Cardiology, Kingston Health Sciences Centre, Queen’s University, Kingston, Ontario, Canada

The authors report no conflicts of interest for the published content. No funding information was provided.

References1.ArominCChandaAKumarSThomasGRA practical ambulatory approach to atrioventricular block secondary to Lyme carditisJ Innov Card Rhythm Manag20231435365536810.19102/icrm.2023.1403136998412PMC100447862.BesantGWanDYeungCSuspicious index in Lyme carditis: systematic review and proposed new risk scoreClin Cardiol2018411216111616[CrossRef]
[PubMed]3035043610.1002/clc.23102PMC64898853.YeungCBaranchukADiagnosis and treatment of Lyme carditis: JACC review topic of the weekJ Am Coll Cardiol2019736717726[CrossRef]
[PubMed]3076503810.1016/j.jacc.2018.11.0354.WangCChackoSAbdollahHBaranchukATreating Lyme carditis high-degree AV block using a temporary-permanent pacemakerAnn Noninvasive Electrocardiol2019243e12599[CrossRef]
[PubMed]3026543210.1111/anec.12599PMC69318325.ModaffDLealMKoppDOutcomes following implant of semi-permanent pacemaker versus temporary pacemakerJ Am Coll Cardiol201769Suppl 1142610.1016/S0735-1097(17)33815-96.WangCNYeungCEnriquezALong-term outcomes in treated Lyme carditisCurr Probl Cardiol20224710100939[CrossRef]
[PubMed]3441703310.1016/j.cpcardiol.2021.100939

The case presentation by Aromin et al.^1^ is an insightful review of a complex case requiring clinical acumen and thoughtful management decisions. As 80%–90% of untreated patients with Lyme disease develop AV dissociation, this case is a crucial learning opportunity for all clinicians taking care of patients with this diagnosis. In this case, the patient was a 31-year-old healthy man presenting with several episodes of syncope and found to be in complete heart block with multiple episodes of ventricular asystole on continuous telemetry monitoring. Other components of the patient’s history, including exposure to a tick, fever, malaise, and rash, led the clinical team to a diagnosis of Lyme disease and LC. The diagnosis was confirmed by serology, and the patient was started on ceftriaxone for empiric management. PPM placement was deferred, and a TPPM was implanted on day 6 of his admission. The patient was discharged home with a TPPM system, and, following completion of his antibiotics, a 48-h Holter monitor and a 12-lead electrocardiogram showed intact AV conduction. The TPPM was removed, and the patient was followed as an outpatient without any further complication.

This case report is an important reminder and example of the importance of conscientious decision-making and careful review of indications when considering PPM implantation. The potential complications of long-term pacing are well established and are particularly relevant in young patients like this one. Indeed, a recent article in *Heart Rhythm* described the many challenges of managing patients with congenital complete heart block requiring long-term pacing. Issues raised in this publication included several generator changes, lead revisions, lead abandonment, and the high prevalence of lead malfunction as well as infection, leading to transvenous lead extraction.^2^ In this study including 16 patients at a high-volume center, a total of 38 leads were removed. Consequently, if contemplating PPM implantation in a young patient, clinicians must also remember the possible serious complications of transvenous lead extraction, which include ventricular laceration, myocardial avulsion, pericardial effusion, hemothorax, and superior vena cava tear necessitating sternotomy.^3^

Given the high likelihood of electrical conduction system recovery in patients with LC and with an in-depth understanding of the risks of long-term pacing, the authors made a thoughtful management decision with implantation of a TPPM and deferment of PPM placement. It is well known among cardiologists and electrophysiologists that traditional temporary pacemaker wires always carry a risk of sudden loss of capture, sensing failure, or dislodgement.^4^ This can lead to critical events in patients with high-grade or complete heart block. Alternatively, TPPM systems have demonstrated greater safety, efficacy, and reliability in patients requiring long-term pacing. In a series of 47 patients published in *Europace* who underwent transvenous lead extraction due to cardiac implantable electronic device infection, patients who were pacemaker-dependent underwent TPPM implantation using a similar technique as that described in this case report. There was no loss of capture or sensing failure in any patient.^5^ In addition to safety and reliability, an active fixation lead allows the patient to be mobile and participate in physical therapy while hospitalized, and it provides greater comfort than a traditional temporary pacemaker wire.

Reversible causes of complete AV block must always be reviewed and ruled out before considering PPM implantation. In addition to LC, another clinical scenario where TPPMs may be a viable option is in the post-transcatheter aortic valve replacement (TAVR) population suffering from conduction system abnormalities. New left bundle branch block and high-degree AV block are common complications encountered post-TAVR; however, recovery of conduction has been shown in >50% of patients who did in fact receive PPMs prior to hospital discharge.^6^ Although the timing of recovery needs to be further investigated in TAVR patients, the case by Aromin et al. is a reminder that we should continue to investigate conduction system recovery in various patient populations before rushing to PPM implantation and exposing patients to other potential complications in the future.

Another crucial point discussed in this case review relates to the cost-effectiveness of TPPM systems. The authors emphasize the high cost of prolonged hospitalizations in critical care units versus the cost of an active fixation endocardial lead and a single-chamber pulse generator. Of note, this case did occur at the peak of the COVID-19 pandemic, when the need for critical care beds was at its highest. Nonetheless, the clinical team had ample resources to safely discharge their patient home with the aid of an assigned nurse specifically trained to care for the pulse generator site and administer intravenous antibiotics. With a traditional temporary pacemaker wire, the patient would have remained hospitalized given the need for continuous monitoring due to the risk of dislodgement and failure to capture. This is certainly an ideal example of care that all health systems should aspire to. Notably, this clinical scenario occurred in a Canadian hospital supported by a universal health care system. It would be of interest to see if a case like this could be replicated or become standard of care in Canada, and if this scenario could be achieved at a reasonable cost to a patient or under coverage by various insurances in the United States. Additionally, as pointed out by the authors in their discussion, this type of discharge requires careful patient selection and family engagement. This scenario may not be feasible in all communities, and hospital stays may in fact be longer with a TPPM system while waiting for conduction system recovery. Nonetheless, regardless of cost or hospital length of stay, allowing AV nodal recovery remains the best clinical decision for our patients and their future.

In summary, successful and careful cardiac implantable electronic device management begins prior to implantation. PPM placement is not always the conclusion to high-grade or complete AV block. The possibility of conduction system recovery should be considered in every case, especially in younger individuals, where long-term pacing will inevitably have consequences and carry a high likelihood of additional procedures and surgeries. In conditions such as LC where most patients do recover electrical conduction, a TPPM system is a viable option that allows for safer, more reliable, and more comfortable pacing than the traditional temporary pacemaker wire. A TPPM system should be considered in conditions other than LC where conduction system recovery is a possibility.

Anne-Sophie Lacharite-Roberge, md^1^ and Ulrika Birgersdotter-Green, md^1^ (ubgreen@health.ucsd.edu)

^1^University of California San Diego, San Diego, CA, USA

The authors report no conflicts of interest for the published content. No funding information was provided.

References1.ArominCChandaAKumarSThomasGRA practical ambulatory approach to atrioventricular block secondary to Lyme carditisJ Innov Card Rhythm Manag20231435365536810.19102/icrm.2023.1403136998412PMC100447862.DardenDBoatengBATsengASTransvenous laser lead extraction in patients with congenital complete heart blockHeart Rhythm202219711581164[CrossRef]
[PubMed]3525797610.1016/j.hrthm.2022.02.0313.SoodNMartinDTLampertRCurtisJPParzynskiCClancyJIncidence and predictors of perioperative complications with transvenous lead extractions: real-world experience with national cardiovascular data registryCirc Arrhythm Electrophysiol2018112e004768[CrossRef]
[PubMed]2945332410.1161/CIRCEP.116.0047684.HynesJHolmesDHarrisonCFive-year experience with temporary pacemaker therapy in the coronary care unitMayo Clin Proc1983582122126
[PubMed]
68231575.KawataHPretoriusVPhanHUtility and safety of temporary pacing using active fixation leads and externalized re-usable permanent pacemakers after lead extractionEuropace201315912871291[CrossRef]
[PubMed]2348261310.1093/europace/eut0456.RaelsonCAGabrielsJRuanJRecovery of atrioventricular conduction in patients with heart block after transcatheter aortic valve replacementJ Cardiovasc Electrophysiol2017281011961202[CrossRef]
[PubMed]2867791710.1111/jce.13291
